# What Dietary Patterns and Nutrients are Associated with Pancreatic Cancer? Literature Review

**DOI:** 10.2147/CMAR.S390228

**Published:** 2023-01-06

**Authors:** Mohammed O Ibrahim, Haya Abuhijleh, Reema Tayyem

**Affiliations:** 1 Department of Nutrition and Food Technology, Faculty of Agriculture, Mu’tah University, Karak, Jordan; 2 Department of Human Nutrition, College of Health Sciences, QU Health, Qatar University, Doha, Qatar

**Keywords:** pancreatic cancer, macronutrients, micronutrients, dietary pattern

## Abstract

This narrative review summarizes the main findings of observational studies (case-control and cohort) as well as systematic reviews and meta-analyses on the role of nutrients and dietary patterns on pancreatic cancer (PC) risk and elucidates possible mechanisms for the association between nutrients or specific food components and the risk of PC. A literature search of MEDLINE (PubMed), Google Scholar, ScienceDirect, and Scopus was performed. An extensive search of related articles published in the English language from 1985 to 2022 was carried out. Our search included macro- and micronutrient intake as well as dietary patterns associated with PC. In conclusion, the consumption of a diet high in nutrients such as sugar, fats, and red and processed meats can increase the risk of PC. Conversely, a high dietary intake of fresh fruit and vegetables and their associated nutrients like fiber, antioxidants, and polyphenols may prevent PC. Dietary patterns loaded with red and processed meats were also linked to an increased risk of PC, whereas dietary patterns rich in plant-based foods like vegetables, fruits, whole grains, and legumes were associated with a reduced risk of PC. Dietary fiber, fat-soluble vitamins, water-soluble vitamins, and minerals might also play a protective role against PC.

## Introduction

Pancreatic cancer (PC) is one of the most rapidly fatal malignancies. Despite being the world’s 12th most common type of cancer, with 495,773 new cases in 2020,^1^ it is the seventh leading cause of cancer-related deaths worldwide, accounting for 4.7% of cancer deaths in 2020.^1^ PC is difficult to diagnose early because there are few signs and symptoms before it spreads beyond the pancreas.^2^ Other than that, the symptoms of PC are similar to those of many other illnesses, making diagnosis difficult, and more than half of the PC patients are diagnosed at a metastatic stage.^3^

Pancreatic cancer is multifactorial and has various risk factors, including gender, age, fat mass, overweight and obesity, smoking, heavy alcohol consumption, a medical history of diabetes and chronic pancreatitis, and genetic predisposition.^4–6^ Diet is a highly correlated risk factor that could be modified to reduce the risk of PC. Several epidemiological studies have examined the associations between individual foods or nutrients, for instance, red and processed meats,^7^,^8^ vegetables and fruits,^9^,^10^ foods rich in fat, sugar, and starch,^6^,^11^,^12^ vitamins and minerals,^13–15^ and fiber^16^,^17^ and the risk of PC, but results are still inconclusive.

A multiethnic cohort of smokers in the United States (US) showed an inverse association between a dietary pattern high in quercetin, kaempferol, and myricetin and the risk of PC.^18^ However, Arem et al found no significant association between flavonoid intake and PC risk.^19^ In the Lowa Women’s Health Study, it included 256 postmenopausal PC women, and the results revealed no significant association between the intake of nutrients and food groups or dietary patterns and PC, and this study did not support the association of fruits, vegetables, or red meat with PC.^20^

Recent studies present substantial controversies, thus, the current review has been directed to discuss the association between nutrient intake and dietary patterns with PC, as well as possible mechanisms for the association between diet and PC risk.

## Methods

We performed a literature search of MEDLINE (PubMed), ScienceDirect, Google Scholar, and Scopus. Our search included macro- and micronutrients intake, as well as different dietary patterns that are associated with the risk of PC. Terms used in the search strategy included the exposures— dietary carbohydrates, carbohydrates-rich diet, sugar and sugar-sweetened soft drink, dietary fibers, high fiber diet, protein-rich diet, red meat and processed meat-rich dietary pattern, high fat diet, water-soluble vitamins, fat-soluble vitamins, major minerals, trace minerals, Mediterranean diet, plant-based diets, vegetable-rich diet, fruit-rich diet—and the risk of PC. Given that the present article included studies from different designs including cross-sectional, case-control, and cohorts studies, as well as systematic reviews and meta-analyses. This article is not a systematic review, and some studies might not have been identified. However, we did an extensive search for related articles published in the English language from 1985 to 2022. All authors conducted the literature search independently. Table 1 shows the summary of the search strategy.

**Table 1:** The Search Strategy Summary

Items	Specification
**Date of Search (specified to date, month and year)**	February–April, 2022
**Databases and other sources searched**	MEDLINE (PubMed), ScienceDirect, Google Scholar, and Scopus
**Search terms used (including MeSH and free text search terms and filters)** **Note: please use an independent supplement table to present the detailed search strategy of one database as an example**	Dietary carbohydrates, carbohydrates rich diet, sugar and sugar-sweetened soft drink, dietary fibers, high fiber diet, protein-rich diet, red meat and processed meat-rich dietary pattern, high fat diet, water-soluble vitamins, fat-soluble vitamins, major minerals, trace minerals, Mediterranean diet, plant-based diets, vegetable-rich diet, fruit-rich diet—and the risk of PC.
**Timeframe**	1985 to 2022
**Inclusion and exclusion criteria (study type, language restrictions etc.)**	Studies from different designs including cross-sectional studies, case-control, and cohorts, as well as systematic reviews and meta-analyses. Articles published in the English language
**Selection process (who conducted the selection, whether it was conducted independently, how consensus was obtained, etc.)**	All of the authors conducted the literature search independently
**Any additional considerations, if applicable**	–

## Nutrients and PC

### Dietary Carbohydrates and PC

The association between high carbohydrate intake and the risk of developing PC was not supported by some studies that were addressing the association between dietary carbohydrates, glycemic load (GL), glycemic index (GI) and the risk of PC.^21^,^22^ In addition, two prospective studies revealed that there was no association between GL, GI, total carbohydrates, total sugar, sucrose, or fructose,^23^,^24^ nor for high sugar foods and PC risk.^25^,^26^ In contrast, Meinhold et al^27^ reported that higher risk for PC was associated with higher percentiles of glycemic load (HR = 1.45, 95%CI: 1.05, 2.00), available carbohydrate (HR =1.47, 95% CI: 1.05, 2.06), and sucrose (HR = 1.37, 95% CI: 0.99, 1.89) intake. This was in line with a prospective study conducted by Michaud et al^28^ indicated that a greater risk of PC was linked to higher dietary GL, GI, and fructose intake in women with a body mass index (BMI) ≥ 25. Moreover, a positive association between sugars and increased risk of PC was reported in a study conducted by Genkinger et al^29^ and Mueller et al.^30^ Likewise, Nöthlings et al concluded from their multiethnic cohort study that high intakes of fructose and sucrose might also play a role in the etiology of PC.^31^

Some studies revealed no association between dietary carbohydrates, glycemic load (GL), glycemic index (GI) and the risk of PC; however, most of the studies emphasized that higher dietary GL plays a significant role as a risk factor for PC.

### Dietary Fibers and PC

According to Bidoli et al,^32^ fiber plays a protective role against the risk of PC. Total fiber intake was found to be inversely related to the risk of PC. In the same context, Zhang et al^33^ and La Vecchia et al^34^ suggested that whole-grain consumption could be considered a protective factor against PC. Moreover, a recent systematic review conducted by Nucci et al 2021 revealed that dietary fiber intake is associated with a reduced risk of PC.^16^ Besides that, an epidemiological study indicated that the risk of developing PC is inversely associated with the intake of fruit and vegetables.^35^ Many factors could explain why certain types of fiber are protective against PC risk. This could be related to fiber’s beneficial effects on insulin metabolism via hormonal pathways linked to insulin-like growth factors, which have been linked to cancer promotion.^36^,^37^ Moreover, the beneficial effects of fiber include its role in reducing insulin resistance and improving glucose tolerance^38^ and this is attributed to decreasing food glycemic index (GI),^39^,^40^ improving subjects’ glucose homeostasis,^39^ and altering gut microbiota.^41^ Dietary fiber through its physicochemical properties plays a crucial role in delaying gastric emptying time and slowing down glucose absorption.^36^ However, Stolzenberg-Solomon et al 2002^42^ found in their cohort study that there was no significant relationship between total intake of soluble or insoluble fiber and PC.

It could be concluded from the studies about dietary fibers and PC, that fibers from different food sources are considered a protective factor in reducing the risk of PC.

### Dietary Proteins and PC

The association between protein intake (animal or plant-based protein) and the risk of PC is critical. In a large prospective cohort conducted by Ghorbani et al, results showed no clear or consistent association between the risk of PC and the intake of dietary protein.^43^ An increased risk of PC was reported by a case-control study to be associated with higher consumption of lamb, veal, and game.^44^ Larsson and Wolk^45^ found that every 50 g of processed meat contributes to a 19% increase in the risk of PC. The findings regarding poultry consumption are highly controversial. Rohrmann et al 2013,^46^ indicated a positive association with PC. However, a negative association has also been revealed by a different prospective study.^47^ In a meta-analysis conducted by Qin et al,^48^ no inverse association was detected between the consumption of fish or long-chain polyunsaturated fatty acids (LC-PUFA) and the risk of PC. They also revealed that only non-fried fish intake was inversely associated with the risk of PC. A recent systematic review and meta-analysis suggested that eating high amounts of may increase the risk of PC, whereas eating high amounts of fish is unlikely to increase the risk of PC.^49^ Moreover, previous research addressed the relationship between grain consumption as a source of dietary protein and the risk of PC and showed positive associations between the intake of refined grains and white bread and the risk of PC.^50–52^ whereas nut consumption was reported to be inversely associated with the risk of PC in a different study conducted by Bao et al.^53^

According to all studies mentioned about the association between dietary proteins and PC, it could be concluded that proteins from refined grains, processed meat, and non-fried fish are considered risk factors for PC. Also, contradicted results regarding poultry and the risk of PC. Finally, nuts may have a protective role from PC.

### Dietary Fats and PC

It has been indicated that the long-term release of cholecystokinin (CCK) upon the presence of lipids in the duodenum, might lead to a high risk of PC.^54^ Some studies reported that the incidence of PC is high in countries that consume high-fat diets.^55–57^ Meanwhile, the incidence of PC is lower in countries that consume diets low in fat.^58^ Several cohort studies have indicated a positive association between the risk of PC and total fat consumption,^59^ saturated fatty acids (SFAs),^42^ and monounsaturated fatty acids (MUFAs).^60^ Nkondjock et al^61^ reported that substituting polyunsaturated fatty acids (PUFAs) with SFAs or MUFAs might reduce the risk of PC, independent of the total energy intake. Another recent cohort study revealed that unsaturated fatty acids including PUFAS, and especially MUFAS, had a protective effect against PC.^62^

Concerning omega-3 and omega-6 PUFAs, opposite associations were shown with the risk of PC in which the risk decreased with the omega-3 fatty acids (FAs)^63^,^64^ and increased with omega-6 FAs.^65^ Intake of other types of fat, such as cholesterol, was significantly associated with the risk of PC.^17^,^66^ In a meta-analysis by Chen et al,^66^ a dose-response relationship was reported with an 8% increase in the risk of PC with every 100 mg/ day intake of cholesterol. Another meta-analysis by Wang et al^17^ found ethnic variability in the association between dietary cholesterol and the risk of PC, in which dietary cholesterol may be associated with the risk of PC in worldwide populations, but not in Europeans. Several mechanisms may explain the possible role of cholesterol in the development of PC. This includes the cellular inflammation that could result from alterations in lipid and apolipoprotein levels^67^ and the high levels of proinflammatory cytokines, such as tumor necrosis factor-alpha (TNF-α) and interleukin-6 (IL-6), which are related to the low levels of high-density lipoprotein cholesterol (HDL-C), high levels of low-density lipoprotein cholesterol (LDL-C) and total cholesterol (TC).^68^

A conclusion from the studies between dietary fats and PC indicated that the high risk of PC is highly attributed to a high-fat diet. Moreover, saturated fatty acids (SFAs), monounsaturated fatty acids (MUFAs), and dietary cholesterol are highly considered risk factors for PC. In contrast, polyunsaturated fatty acids (PUFAs) play an excellent role as protective factors for PC.

### Water-Soluble Vitamins and PC

In a study conducted by Bravi et al in 2011, there was a clear protective role for thiamin, riboflavin, folate, and vitamin C.^13^ Multiple logistic regression models were developed to compare the highest and lowest quintile of intake, and results revealed a strong association with an Odd Ratio of ((OR) = 0.73, 0.73, 0.56, and 0.44) for thiamin, riboflavin, folate and vitamin C, respectively. The vitamin C results were in line with a case-control study conducted by Ji et al 1995^69^ which revealed the same association. The protective role of vitamin C against PC is explained through its role in reactive oxygen species,^70^ autophagy^71^ and depletion of intracellular adenosine triphosphate.^72^ Moreover, a prospective nested case-control study indicated an inverse relationship between circulating levels of vitamin B_12_ and B_6_ and the risk of PC.^73^ In addition, a recent meta-analysis conducted by Wei1 and Mao^74^ examined the association between the risk of PC and the intake of vitamin B_6_ and vitamin B_12_. They reported a 9% decrease in the risk of PC for every 10 nmol/L increments in blood pyridoxal 5′-phosphate (PLP) levels, but there was no significant association between the risk of PC and vitamin B_12_ intake.^74^ On the other side, Gong et al 2009^75^ suggested that a higher intake of vitamin B_12_ is associated with an increased risk of PC. Vitamin B_6_ plays a protective role in the development of PC.^76^ This is because it is a cofactor in DNA synthesis, and is involved in the methylation pathway of one-carbon metabolism.^76^ Low intake of vitamin B_6_ reduces the production of methylene- THF (methyl donor) which could lead to hypomethylation and promote oncogenesis.^77^

Generally concluded for the studies regarding the risk of PC and WSV, it is noted that vitamins such as B_1_, B_2_, B_6_, B_12_, folate, and vitamin C have a distinguished protective role from PC.

### Fat-Soluble Vitamins and PC

Obvious conflict in the studies investigating the association between PC and fat-soluble vitamins has been documented.^13^,^73^,^74^ Bravi et al^13^ conducted an analysis comparing the highest to the lowest quintile of ß –carotene intake and the results showed no significant inverse relationship between the risk of PC and the intake of ß –carotene. On the other hand, a case-control study found an inverse relationship between ß-carotene and the risk of PC.^78^ Meanwhile, another study found no association.^79^ The most common mechanisms underlying the protective role of vitamin A in PC include retinoic acid receptor modulation,^80^ interaction with protein kinase,^81^ inhibition of cellular adhesion,^82^ and downregulation of IL-6.^83^ Two prospective cohort studies were conducted to investigate the association between vitamin D intake and PC risk.^84^,^85^ They found that a lower risk of PC was associated with a higher intake of vitamin D (≥ 600 IU per day). In contrast, a case-control study revealed that a higher intake of vitamin D (≥ 450 IU/day was associated with an increased risk of PC in men.^86^ However, a meta-analysis reported that dietary vitamin D is not associated with the risk of PC.^87^ Five common underlying mechanisms reveal the anti-pancreatic cancer effect of vitamin D. These mechanisms include vitamin D receptor agonist,^88^ AMP-activated protein kinase (AMPK)-dependent mechanisms,^89^ inhibition of phosphatidylinositol 3-kinases (PI3K)/protein kinase B (AKT) pathway,^90^ regulation of the cell cycle,^91^ and decreased cell migration and invasion.^92^ The protective role of vitamin E was clear in the study conducted by Bravi et al 2011.^13^ They demonstrated that after comparing the highest to the lowest quintile of intake, for vitamin E (OR= 0.60, 95% confidence interval (CI): 0.36–0.98). Another case control by Ji et al^69^ revealed the same associations. Mechanisms behind this association include the protection of vitamin E against PC through its role in the inhibition of nuclear factor-kB (NF-kB) activity,^93^ Ras-Raf-MEK-ERK pathway,^94^ regulation of the cell cycle,^95^ and induction of apoptosis.^96^ Previous studies indicated that vitamin K might act as a potential antitumor agent. From a metabolic point of view, vitamin K has a protective role from PC through its ability to suppress cancer growth and induce apoptosis and differentiation in PC cells.^97^,^98^

The studies mentioned regarding FSV and PC in general give important indicators that vitamin A, vitamin D, vitamin E, and vitamin K are having magical mechanisms behind their role as protective factors from PC.

### Major Minerals and PC

A recent case-control study conducted by Fan et al 2021^99^ indicated that the intake of calcium and phosphorus was significantly lower among cases compared to controls. Moreover, they found that there were no significant associations between the total intake of calcium and phosphorus with the risk of PC.^99^ In another case-control study, they reported that odds ratios of PC risk increased with higher calcium intake among men but not in women.^86^ In a prospective study conducted by Kesavan et al 2010,^100^ a 33% reduction in the risk of PC was associated with magnesium supplements in the study group. The protective role of both sodium and potassium against the risk of PC was emphasized in a study conducted by Bravi et al 2011.^13^ They found that comparing the highest to the lowest quintile, the OR significantly reduced (OR=0.57, 95% CI: 0.35–0.92) for potassium intake, while the increase in the OR was insignificant for sodium intake.

Upon the results of studies between major minerals and PC, it is concluded that calcium, phosphorus, magnesium, sodium, and potassium are highly considered protective factors against PC. An exception was found regarding calcium because gender may play a major role in the risk of PC with higher intakes of calcium among males.

### Trace Minerals and PC

An exploratory analysis in the US discovered no association between iron intake and the risk of PC.^56^ This finding was also reported by the European Prospective Investigation into Cancer and Nutrition (EPIC) cohort on studying the association between total iron and heme-iron and the risk of PC.^101^ The meta-analysis conducted by Li and Gai^102^ indicated the protective role of zinc against the risk of PC. In contrast, Bravi et al 2011^13^ reported zinc intake is not associated with the risk of PC. Most of the studies confirm the significant role of chromium in enhancing insulin action and improving glucose tolerance and such roles require a healthy pancreas.^103^ Another study indicated the antioxidant capabilities of chromium in reducing oxidative stress.^104^ Epidemiological studies found a relationship between thyroid dysfunction and pancreas pathology and that thyroiditis could increase the risk of PC.^105^ Therefore, adequate iodine intake is associated with normal thyroid hormone levels and normal pancreatic function which may reduce the risk of PC.^106^ Several studies indicated that a high intake of selenium was associated with a reduced risk of PC.^107–109^ Finally, some trace minerals such as lead, cadmium and arsenic were significantly associated with increased risks of PC in the highest quartile.^107^ Table 2 summarizes the findings of these studies.

**Table 2:** Summarization of the Studies on Trace Minerals and PC

Trace Minerals	Findings
**Iron**	In an exploratory analysis in the US, it was found that there is no association between iron intake and the risk of PC.^56^ This was also reported by the European Prospective Investigation into Cancer and Nutrition (EPIC) cohort on studying the association between total iron and heme-iron and the risk of PC.^101^
**Zinc**	Li and Gai^97^ conducted a meta-analysis that included seven studies to investigate the association between zinc intake and the risk of PC. Their results indicated the protective role of zinc against the risk of PC. In addition, they found that the risk of PC significantly decreased in the group with the highest zinc intake as the PC risk for the highest versus the lowest categories of zinc intake was (RR= 0.798, 95% CI: 0.621–0.984).
**Chromium**	Most of the studies confirm the significant role of chromium in enhancing insulin action and improving glucose tolerance and such roles require a healthy pancreas.^98^ Another study indicated the antioxidant capabilities of chromium in reducing oxidative stress.^99^
**Iodine**	Epidemiological studies found a relationship between thyroid dysfunction and pancreas pathology and that thyroiditis could increase the risk of PC.^100^ Therefore, adequate iodine intake is associated with normal thyroid hormone levels and normal pancreatic function which may reduce the risk of PC.^101^
**Selenium**	Several studies indicated that a high intake of selenium was associated with a reduced risk of PC.^102–104^
**Lead, cadmium and arsenic**	Lead, cadmium and arsenic were significantly associated with increased risks of PC in the highest quartile.^102^
**Nickel**	Nickel was inversely associated with the risk of PC with (OR = 0.27, 95% CI: 0.12 to 0.59).^102^

The data collected regarding the associations between trace minerals and the risk of PC concluded that most of these minerals play a protective role while minors are considered as risk factors and others have no association. Zinc, chromium, iodine, selenium, and nickel represent the protective minerals while lead, cadmium and arsenic represent the risk factor minerals. No association was indicated between total iron and heme-iron and the risk of PC.

## Dietary Patterns and PC

### Mediterranean Dietary Pattern and PC

Mediterranean diet (MD), which is characterized mainly by high consumption of plant-based foods, has been linked to a decreased risk of various types of cancer. However, the evidence regarding its relationship with PC remains inconclusive. An Italian case-control study investigated the association between the MD and the risk of PC,^110^ and reported a decrease in PC risk by 15% per one-unit increase in adherence to the MD. Moreover, they found consistency in this association across sub-group analysis of age, BMI, smoking status, and alcohol consumption and it was stronger among non-diabetic participants compared to diabetics (OR= 0.84 and OR=0.99 respectively, P for interaction=0.01). The National Institutes of Health-AARP Diet and Health Study evaluated this association prospectively in diabetes-free individuals and included cases of PC.^111^ Adherence to the MD on of the components of the healthy lifestyle score which also considered other factors such as tobacco use, alcohol drinking, BMI, and physical activity. According to this study, those who scored the highest on the lifestyle score had a significantly lower risk of PC compared to those who scored lowest (Relative risk =0.42, 95% CI: 0.26–0.66).^111^ A plausible mechanism that can explain the role of MD as a cancer-preventive diet is based on the suppression of inflammation and carcinogenic pathways. This has been attributed to the powerful effect of nutritional components of the MD diet, which include healthy fats (omega 6 and omega 3), fibers, antioxidants, fruits, vegetables, legumes, and olive oil.^112^

However, this is inconsistent and limited among available studies. In a large European prospective cohort study,^113^ adherence to a non-alcohol-defined Mediterranean diet score (arMED) was not associated with PC risk, nor was there evidence of a significant association between the arMED score and risk of PC in stratified analyses by diabetes, smoking status, BMI, or European region. Moreover, A meta-analysis was conducted to investigate the association between adherence to the Mediterranean diet and the overall risk of cancer. The study results showed that colorectal cancer risk could decrease by 14% and prostate cancer risk could be reduced by 4%. However, the decrease in risk for breast, gastric, or PC was not significant.^114^ This was in line with a pooled analysis of two Dutch cohorts, which found no significant association between adherence to the Mediterranean diet and the risk of PC.^115^

### Dietary Pattern Rich in Vegetables and Fruits and the Risk of PC

**Figure 1:**
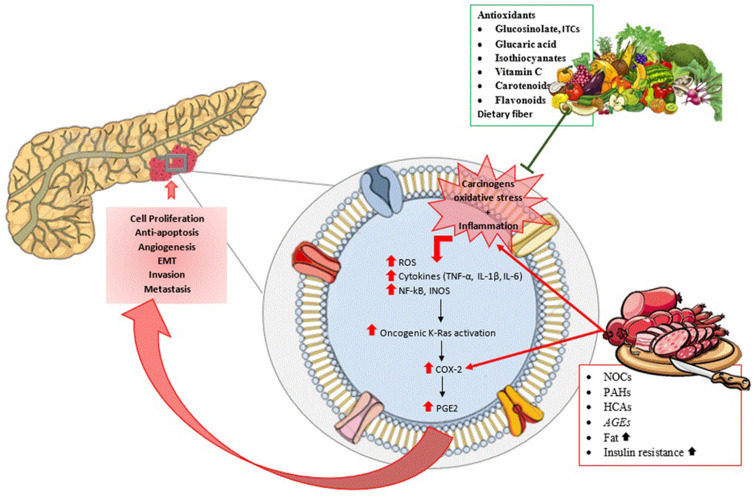
Carcinogens, oxidative stress and inflammation can trigger inflammatory pathways that promote cell proliferation, anti-apoptosis, angiogenesis, epithelial-to-mesenchymal transition (EMT), invasion and metastasis. The presence of antioxidant nutrients (carotenoids and vitamin C), dietary fiber, and other phytochemicals (eg, flavonoids) provide a potential explanation for the protective effect of fruits and vegetables on PC by preventing the carcinogens, oxidative stress, and inflammation which initiate cancer pathogenesis. Cruciferous vegetables specifically have other potent antioxidants such as glucosinolate, indole-3-carbinol (ITCs), glucaric acid, and isothiocyanates. On the other hand, mechanisms that link red or processed meat to pancreatic carcinogenesis, include by-products of cooking methods such as polycyclic aromatic hydrocarbons (PAHs), heterocyclic amines (HCAs), N-nitroso compounds (NOCs), advanced glycation end-products (AGEs), increased fat intake, and insulin resistance.

The majority of PC risk factors are thought to cause oxidative stress, activate inflammation, and, eventually result in high molecular alterations, which could lead to the prognosis of PC^116^,^117^ (Figure 1). Fruits and vegetables, which are high in antioxidants and anti-inflammatory nutrients, could result in a decrease in oxidative stress and inflammation.^118^

A case-control study found that the majority of the nutrients in fruits and vegetables, both dietary and in supplements, are associated with a lower risk of PC.^119^ In this study, cases were more likely than controls to be older, males, non-smokers, and with a history of pancreatitis or diabetes. In other words, when compared to their controls, the cases had high-risk factors that have been consistently associated with PC risk, yet the consumption of fruits and vegetables was effective.^119^ The mechanisms of fruits and vegetables’ cancer-preventive effects could be due to various factors. The presence of antioxidant nutrients (eg, carotenoids and vitamin C), dietary fiber, and other phytochemicals (eg, flavonoids) provide a potential explanation for this protective effect.^120^ Vitamin C reduces free radicals and reactive oxygen molecules, protecting against oxidative damage while inhibiting carcinogen formation and shielding DNA from mutagenic attack.^12^ Also, flavones have been shown to inhibit the cell process linked to carcinogenesis.^12^ In a prospective Japanese study,^9^ they studied the relationship between fruit and vegetable consumption and PC and found that total fruit intake was negatively associated with PC risk and positively associated with total vegetable intake in every smoker. However, vegetable consumption may be associated with an increased risk due to the impact of smoking status on vegetable consumption.^9^

A hospital-based, case-control study included 183 PC patients and 732 to study the association between cruciferous vegetables (ie, broccoli, cauliflower, cabbage, and Brussels sprouts) and PC,^121^ and used a self-administered questionnaire to collect data. They found that usual intakes of raw cruciferous vegetables were negatively related to PC. where the highest level of raw cruciferous vegetable intake was associated with a 40% lower risk of PC in the study participants, and a 50–59% lower risk in groups that included non-modifiable risk factors, such as previous smokers, overweight participants, and males.^121^ This study supports the powerful anti-carcinogenic effect of cruciferous vegetables acting against PC.

Patients with pancreatic adenocarcinoma had significantly elevated levels of total unconjugated bile acids.^122^ One suggested plausible mechanism is related to the release of bile acid, in conjunction with a long common channel of the biliary and pancreatic ducts, which could result in bile acid reflux into the pancreatic duct and to the epithelial, or acinar, cells that give rise to pancreatic adenocarcinoma.^123^ Moreover, bile acids increase the expression of cyclooxygenase-2 (COX-2) in PC cells.^124^ COX-2, an enzyme that catalyzes prostaglandin synthesis, is known to be overexpressed in pancreatic adenocarcinoma.^124^ Cruciferous vegetables have properties that may help the bile production process. In an in-vitro study, cruciferous vegetables, indole-3-carbinol (ITC) and glucaric acid have bile acid binding properties and are powerful antioxidants and inducers of certain enzymes that act as detoxifying agents of harmful metabolites and bile acids, consequently inhibiting mechanisms of bile acid that potentially could result in PC.^121^ These vegetables also contain phytochemicals which help prevent PC. Glucosinolate is a dietary ITC precursor that has been shown to preserve chemically induced tumors by regulating epigenetic mechanisms.^121^ ITC and some isothiocyanates, compounds found in cruciferous vegetables, such as sulforaphane, benzyl isothiocyanate, and phenethyl isothiocyanate, have been shown in animal studies to have a suppressive effect on cancer cells of the pancreas.^125^

Despite that many case-control studies have found an inverse association between the consumption of fruits and vegetables and the risk of PC, A meta-analysis of prospective studies found that consuming large amounts of fruits and vegetables citrus fruit or cruciferous vegetables was not associated with a lower risk of PC.^10^ It was also stated that, while increased consumption of fruits is associated with a lower PC risk in men but not in women, the studies included are limited.^10^ Furthermore, dose-response analyses revealed no significant dose-response relationships between an increase in fruit and vegetable consumption of 100 g/d and PC risk. Moreover, the results of subgroup and subtype analyses were consistent with the results of the original analyses.^10^ Concluding that prospective study analyses revealed no evidence of the relationship between fruit and vegetable consumption and the risk of PC.

Overall, the association between the consumption of fruits and vegetables and the risk of PC is inconclusive. A better understanding and further examination of this association are required.

### Dietary Patterns Rich in Red and Processed Meat and the Risk of PC

Based on a review of mechanistic and human evidence for colorectal cancer, the International Agency of Research on Cancer (IARC) has classified red and processed meats as probable and definite carcinogens, respectively.^126^ Yet, the association between red and processed meat and PC is very limited and inconclusive. According to a systematic review and meta-analysis, there was an association between the consumption of red and processed meat and the risk of PC in case-control studies but not in cohort studies.^8^ More recently, Petrick et al 2020^7^ found a positive association between red and unprocessed red meat and the risk of PC in African American women with a mean age of 50 years, but not among women of a younger age. Consumption of red and processed meat, on the other hand, appeared to increase the risk of PC in men but not in women in cohort studies. In another meta-analysis conducted on studies investigating the association between red and processed meat intake and the risk of PC,^45^ it was found that consumption of processed meat has a statistically positive association with the risk of PC. A 50 g increase in processed meat consumption per day was linked to a 19% increased risk of PC. While consumption of red meat was only positively associated with PC in men, that was explained by suggesting a threshold effect in red meat consumption which could be detected only at higher amounts usually achieved by men than in women on average. However, in a longitudinal study done on African American women, higher intake of red meat compared to lower intake was associated with a 66% increase in the risk of PC in 50 years old women and older, while no significant association was found with the consumption of processed meat. In women aged 50 and older, intake of saturated fat was associated with an increase in the risk of PC, but the findings were not statistically significant.^7^

Several plausible mechanisms have been suggested to link red or processed meat to increased carcinogenesis in the pancreas, which includes the consumption of by-products of cooking such as polycyclic aromatic hydrocarbons, heterocyclic amines, N-nitroso compounds, advanced glycation end-products,^127^,^128^ and increased insulin resistance.^129^ Several studies aimed at investigating mutagens formed in meats as a result of grilling, barbecuing, or high-temperature cooking that increases the mutagenic activity in the body and leads to an increased risk of PC.^45^,^130^ In experimental models, N-nitroso compounds are known to be potent carcinogens,^131^ and tobacco smoking is a well-known risk factor for PC.^132^ Other than that, exposure to N-nitroso compounds could be through food consumption. N-nitrosamines form specifically in meat that has been preserved with nitrates, such as cured, smoked, and pickled meat, or meat that has been dried at high temperatures, which adds to the endogenously formed N-nitroso compounds that form in the stomach by the ingestion of nitrate and amides found in meat.^133^ Through the bloodstream, both consumed and endogenously formed N-nitroso compounds reach the pancreas and act as potent carcinogens there.^7^ Moreover, saturated fat and red and processed meat are linked to increased insulin resistance, which can lead to type 2 diabetes. Pre-existing type 2 diabetes is linked to an increased risk of PC.^129^

### Dietary Patterns Rich in Sugar and Sugar-Sweetened Soft Drink Consumption and Risk of PC

Diabetes mellitus patients are at a higher risk of PC.^134^ Researchers found a dose-response relationship between PC risk and fasting glucose levels even in populations with a normal range of blood glucose,^135^ supporting a causal relationship between the two variables. Another study found an association between post-load serum glucose concentration and the risk of PC. Thereby, supporting the association of the role of impaired glucose intolerance, insulin resistance, and hyperinsulinemia in PC.^136^,^137^ Furthermore, a high glycemic load diet has been linked to an increased risk of diabetes and PC.^136^

Sugar-sweetened soft drinks are high in fructose corn syrup, which quickly increases serum glucose levels and consequently increase the risk of diabetes upon frequent consumption. Soft drinks deliver the greatest amounts of added sugar in the diet in America, adding to the high glycemic index content of the diet and driving the development of obesity and diabetes.^138^ Increased consumption of sugar-sweetened beverages was linked to an increase in weight gain and risk of type 2 diabetes in previous research of Nurses’ Health Study (NHS) participants, regardless of the risk factors.^138^ Because of their readily absorbable carbohydrates, sugar-sweetened soft drinks have been linked to an increased risk of type 2 diabetes. Sweetened soft drinks may contribute to the overall diet’s high glycemic load, which is a risk factor for PC.^136^ Furthermore, cola soft drinks have caramel coloring, which is high in advanced glycation end products, which may worsen insulin resistance and inflammation.^138^

On the other hand, a systematic review and meta-analysis conducted on dietary fructose, carbohydrate, glycemic indices, and risk of PC found that high glycemic index, glycemic load, total carbohydrates, or sucrose diets are not associated with the risk of PC. However, they reported an association between the consumption of fructose and an increased risk of PC.^22^ The exact mechanism underlying the association between fructose consumption and PC is unknown, but fructose metabolism differs from that of other carbohydrates such as glucose. Fructose contributes more to nucleic acid synthesis than glucose through the pentose phosphate pathway, which is catalyzed by transketolase.^139^ The synthesis of nucleic acids and nucleotides is required for the proliferation of tissues, particularly cancer cells. Transketolase-like protein 1 suppression reduces cancer cell proliferation, whereas transketolase activation promotes tumor growth.^139^ Furthermore, animal studies have shown that chronic fructose feeding causes insulin resistance and obesity.^140^ A positive relationship was found between fructose consumption and type 2 diabetes and obesity, establishing risk factors for PC.^141^ However, this association remains inconsistent and requires further investigation.

## Conclusion

In summary, this narrative review showed inconsistent findings for associations between nutrient intake and dietary patterns and PC risk. The high intake of sugar, fructose, and fat may increase the risk of PC, whereas the consumption of dietary fiber, fat-soluble vitamins, water-soluble vitamins, and minerals might play a protective role against PC. Dietary patterns characterized by high consumption of red and processed meats, sugar, and sugar-sweetened soft drinks were associated with an elevated risk of PC, and dietary patterns rich in plant-based foods, vegetables, fruits, whole grains, legumes, and white meat and associated antioxidants and polyphenols reduced the risk of PC. Further studies are warranted to assess the potential anticarcinogenic role of specific foods against PC because the diet could have an impact on the development of PC. More studies are needed to verify and clarify the underlying reasons for the gender disparities in the association between nutrient intake and dietary patterns with PC risk.
